# Towards a better understanding of physical activity in people with COPD: predicting physical activity after pulmonary rehabilitation using an integrative competence model

**DOI:** 10.1177/1479973121994781

**Published:** 2021-03-11

**Authors:** Johannes Alexander Carl, Wolfgang Geidl, Michael Schuler, Eriselda Mino, Nicola Lehbert, Michael Wittmann, Konrad Schultz, Klaus Pfeifer

**Affiliations:** 1 Department of Sport Science and Sport, Friedrich-Alexander-University Erlangen-Nürnberg, Erlangen, Germany; 2 Institute for Clinical Epidemiology and Biometry, University of Würzburg, Würzburg, Germany; 3 Klinik Bad Reichenhall, Centre for Rehabilitation, Pulmonology and Orthopaedics, Bad Reichenhall, Germany; *These authors contributed equally.

**Keywords:** Physical activity, pulmonary rehabilitation, chronic obstructive pulmonary disease, health behaviour, physical activity-related health competence

## Abstract

The integrative Physical Activity-related Health Competence (PAHCO) model specifies competences (movement competence, control competence, and self-regulation competence) that enable people to lead a physically active lifestyle. This longitudinal study analyses the predictive quality of a multidimensional PAHCO assessment for levels of physical activity (PA) and their relevance for quality of life in COPD patients after pulmonary rehabilitation. At the end of an inpatient pulmonary rehabilitation (T2), 350 COPD patients participating in the Stay Active after Rehabilitation (STAR) study underwent assessments, including a six-factor measurement of PAHCO. PA (triaxial accelerometry) and quality of life (Saint George’s Respiratory Questionnaire) were recorded 6 weeks (T3) and 6 months (T4) after rehabilitation. Structural equation modelling (SEM) was used to regress the PAHCO assessment on PA, which should, in turn, influence quality of life. In univariable analysis, five and six factors of the PAHCO model were related to PA and quality of life, respectively. Multivariate modelling showed that the predictive analyses for the PA level were dominated by the 6-minute walking test representing movement competence (0.562 ≤ |β| ≤ 0.599). Affect regulation as an indicator of control competence co-predicted quality of life at T3 and levels of PA at T4. The PA level was, in turn, significantly associated with patients’ quality of life (0.306 ≤ |β| ≤ 0.388). The integrative PAHCO model may be used as a theoretical framework for predicting PA in COPD patients following pulmonary rehabilitation. The results improve our understanding of PA behaviour in COPD patients and bear implications for person-oriented PA promotion.

## Introduction

Pulmonary rehabilitation (PR) is an evidence-based treatment for patients with chronic obstructive pulmonary disease (COPD) that provides clinically important short-term benefits for dyspnoea, fatigue, exercise capacity, and quality of life.^1^ After the completion of PR, the achieved benefits begin to diminish if patients do not continue to exercise regularly.^2^ Physical activity (PA) levels in patients with COPD are often low,^3–5^ leading to poor prognosis and negative health outcomes.^6^ Regular PA is recommended at all stages of COPD due to its positive effects on symptoms, exercise capacity, quality of life, risk of hospitalization, and mortality risk.^7^ Despite a wide interest in interventions and strategies for promoting active lifestyles in patients with COPD,^8,9^ the effect of PR on PA levels is still limited.^10^ Thus, promoting PA and maintaining it in the long-term remains a challenge for PR.

The low efficacy of PR regarding PA promotion might be attributed to a limited understanding of the determinants of PA maintenance following rehabilitation.^11,2^ In general the determinants of PA behaviour are multi-faceted and include personal, physiological and psychological elements as well as social and environmental factors.^12^ In patients with COPD, exercise capacity has often been regarded as the central factor influencing PA behaviour.^13^ However, improved exercise capacity does not automatically lead to increased PA levels,^14,15^ as these are not determined by disease-related functional characteristics alone. Psychological theories of PA behaviour^16^ and the patients themselves^11^ report, amongst others, several psychological constructs that influence PA, including PA-related intentions, self-efficacy, and self-perceptions. What has been missing so far is a model that integrates the functional, disease-related aspects *and* the important psychological constructs to better understand PA behaviour in patients with COPD.

The Physical Activity-Related Health Competence (PAHCO) model provides an integrative understanding of person-related determinants that facilitate adherence to health-enhancing PA.^17,18^ The model integrates various abilities and skills that enable individuals to lead a healthy, physically active lifestyle. From a conceptual-historical standpoint, PAHCO follows calls of the educational sciences to promote the competence orientation and calls of rehabilitation science and sport science to integrate different disciplinary perspectives on the topic of PA promotion (e.g., exercise science, human movement science, exercise psychology).^18^

**Figure 1.**
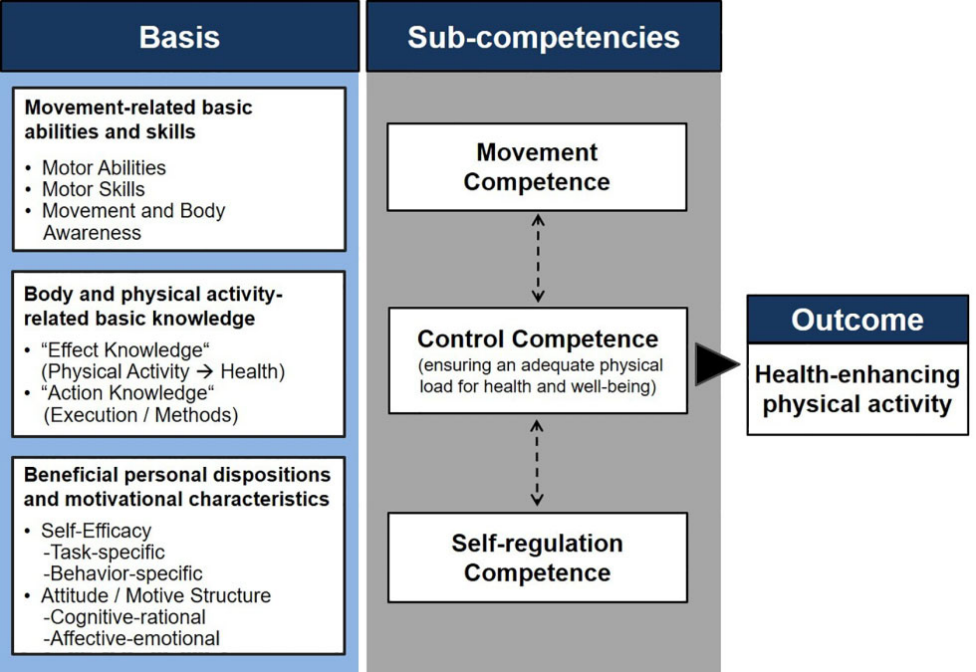
The model of physical activity-related health competence (PAHCO).^17^

The PAHCO model (Figure 1) holds that individuals need three central sub-competencies for a healthy, physically active lifestyle.^17^ First, people need *movement competence*, which, defined as the direct motor-related requirements for PA, helps individuals ‘perform physical activities with vigor and promote resistance to fatigue’.^19^ Second, individuals require *self-regulation competence*, which guarantees regular PA through the generation of motivational-volitional power. The third area, *control competence*, ultimately ensures that individuals do not merely apply any physical stimulus as frequently and intensively as possible. Instead, covering the qualitative side of health-enhancing PA, this dimension refers to the adequate alignment of physical loads in terms of physical health^19^ and subjective well-being.^20^

Although the PAHCO framework has been developed as a generic model, it may be applied particularly in the context of noncommunicable diseases such as COPD^21^ because patients with COPD often show characteristics indicating lower movement compentence,^22^ decreased control competence,^23,11^ and reduced self-regulation competence.^24,11^

### Purpose of this study and hypotheses

The overarching goal of the Stay Active after Rehabilitation (STAR) study was to gain a better understanding of PA in patients with COPD before and after undergoing inpatient PR.^25^ The present article addresses the second main research question of the STAR study, which, in line with the corresponding study protocol,^25^ specifically concentrates on the measurement time points after PR (T2–T4) and examines the relevance of PAHCO in predicting PA after PR.

**Figure 2.**
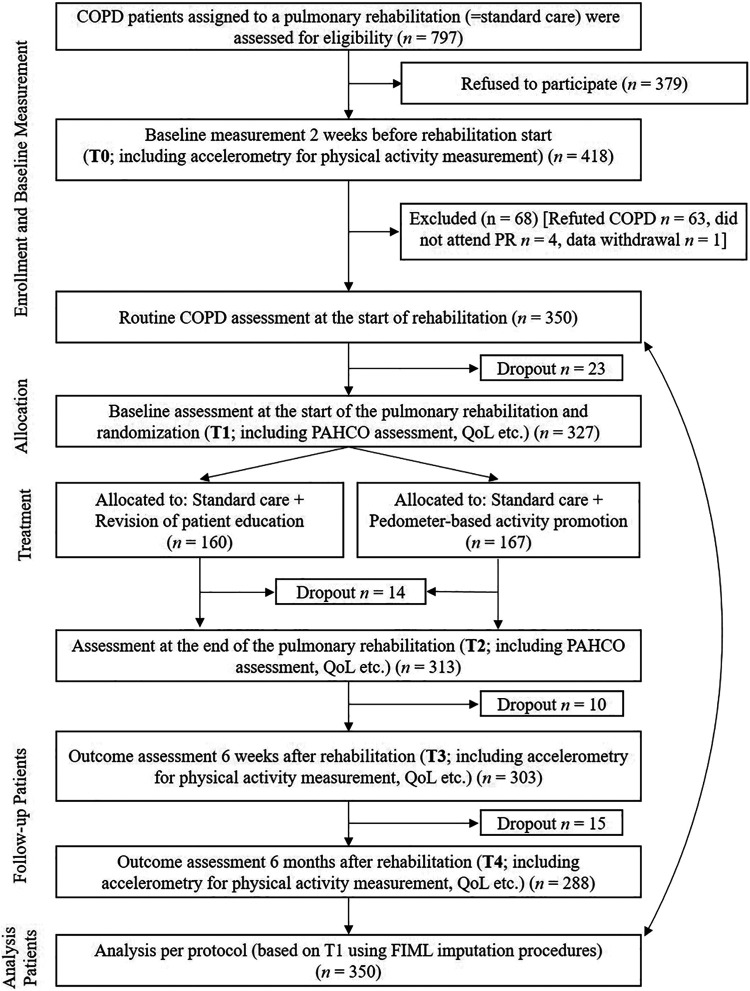
Study flow chart.

The STAR study used a randomised, controlled trial (RCT) design with two parallel groups and five measurement points: T0 = 2 weeks before the start of inpatient rehabilitation; T1 = the start of the rehabilitation; T2 = the end of the rehabilitation; T3 = 6 weeks and after rehabilitation; and T4 = 6 months after rehabilitation (for the study flow chart, see Figure 2). The longitudinal character of the STAR study allowed us to conduct the following analyses including all study participants (i.e., those in both the intervention and the control group): First, we examined how the multivariate PAHCO assessment at the end of the rehabilitation stay (T2) predicted the amount of PA 6 weeks (T3) and 6 months (T4) after rehabilitation. Since the PAHCO model incorporates health-enhancing PA as a primary outcome, we included patients’ quality of life as a second dependent variable. The PA level should, in turn, significantly influence quality of life. We hypothesized that (a) at least one indicator of each PAHCO sub-competence (movement, control, self-regulation competence) would significantly predict the amount of PA at both T3 and T4. Against the background of a recent validation study,^19^ we specifically assumed that the 6-minute walking test (representing movement competence as a proxy indicator), the self-control factor (representing self-regulation competence), and the affect regulation factor (representing control competence) presented the dominant predictors in multivariate models. As control competence yields a health-enhancing impact beyond the mere level of PA we (b) expected an independent, direct effect from one control-competence factor on quality of life. In line with the assumed temporal stability of competences and traits in general and their potential to predict behaviour in the short and middle term, we hypothesized finally that (c) the predictive power of PAHCO at T2 was stronger for the two outcomes when measured at T3 than at T4.

## Methods

### Participants and study design

Figure 2 displays the design of the randomised, controlled STAR study (Clinical Trials Registration Number Clinicaltrials.gov, ID: NCT02966561). The STAR study took place within the German rehabilitation system which typically provides an inpatient rehabilitation in a disease-specific rehabilitation clinic for a duration of three weeks. All study participants received a comprehensive, multidisciplinary PR based on an initial assessment followed by a combination of patient-tailored therapies. Exercise therapy is the highest volume intervention in German PR. All study participants received 4–5 units/week of endurance training, 3 units/week of strength exercise, and 7 units/week whole-body vibration muscle training. In addition, the intervention group received a brief pedometer-based PA promotion intervention. The study protocol describes in detail the methodology of the STAR study.^25^ Here, we outline only the methodological aspects that are crucial for the present research questions. A total of 797 patients were contacted by telephone prior to their PR stay. Among them, 418 persons gave initial consent to participate. A further 68 participants had to be excluded for the following reasons: (a) the COPD diagnosis (initially made by a general practitioner) could not be confirmed by a lung function test (Tiffeneau index FEV1/VC not ≤0.70) that was regularly performed at the beginning of the PR (*n* = 63); (b) the planned clinic stay could not be realized (*n* = 4); or (c) the consent to data use was withdrawn after T0 (*n* = 1). All remaining 350 individuals were subject to data analysis, irrespective of the group assignment (i.e. to the intervention or control group) and their later potential dropout (*n* = 62). A baseline description of the sample (T0/T1) is contained in Table 1, while the most important parameters after rehabilitation (T2) and their change scores across the rehabilitation program can be retrieved from Supplementary File 1.

**Table 1.** Description of the sample at baseline (T0/T1).

Variable	Description
Sample size	*n* = 350
Gender	68.5% male, 31.5% female
Age	58.17 ± 5.56 years
Body mass index (kg/m^2^)	27.64 ± 6.76
FEV_1_ (%)	53.51 ± 18.36
Severity of the disease 1/2/3/4 (Functional GOLD Degree) (%)	8.7/45.1/37.3/9.0
GOLD Symptom and Risk Groups A/B/C/D (%)	1.6/43.6/0/54.8
Saint George’s Respiratory Questionnaire (SGRQ) score	52.64 ± 10.62
Number of comorbidities	4.74 ± 2.43
Number of steps (per day)	5,809 ± 3,054
6-Minute walking test (m)	447.15 ± 104.24
Employment status (%)	60.8% full-time, 14.5% part-time, 15.8% no employment, 9.0% pension
Percentage of smokers (%)	46.7% current smokers, 50.6% former smokers, 2.7% no smokers

### Measures

The level of PA was measured objectively with the validated triaxial ActiGraph (Pensacola, Florida) wGT3X-BT accelerometer.^26^ The participants were advised to wear the accelerometer during their daily routine for 7 days for at least 10 hours a day. A measurement was considered valid if the patients had a wear-time of ≥10 h per day for at least five of the seven measuring days with no requirements for specific numbers of weekend or week days. The technical settings and data processing followed COPD-specific recommendations^27^ and were recently published in detail.^5,25^ To determine the level of PA, we drew on the number of daily steps, which has high clinical relevance in patients with COPD.

PAHCO was assessed with six factors using a combination of questionnaire-based self-reports and a physical function test. Sudeck and Pfeifer^17^ developed a three-dimensional assessment instrument for PAHCO. This questionnaire includes the two factors control of physical load and affect regulation, covering the dimension of control competence. The *control of physical load* factor (6 items, Cronbach’s *α* = .85) refers to patients’ abilities to align the load of activities in such a way that they benefit their physical health (e.g., disease-adjusted training). The *affect regulation* factor (4 items, *α* = .88) quantifies how well patients are able to gear PA toward positive affective reactions and subjective well-being. The self-regulation competence is represented by self-control (3 items, *α* = .85), which quantifies how well individuals succeed in turning activity-related intentions into action.^17^ Within the scope of a stepwise extension strategy, this instrument was recently validated and expanded to five factors by operationalizing two further facets of self-regulation competence: *PA-specific self-efficacy* (3 items, *α* = .82) and *emotional attitudes toward PA* (3 items, *α* = .94).^19^ This validation study revealed satisfactory psychometric quality criteria of the generic PAHCO questionnaire in patients with COPD. The item wording can be found in Supplementary File 2; the Supplementary File 3 contains the correlations between the different PAHCO factors. Since at the time of the STAR study no specific, validated self-report instrument was available for assessing movement competence, we used a *six-minute walking test* ^28^ as a proxy measure. In this test, the participants were advised to cover as much distance as possible in a standardized, flat area. The number of meters achieved within 6 minutes served as the criterion for movement competence.

The patients’ *quality of life* was operationalized via Saint George’s Respiratory Questionnaire.^29^ We used the total score of the validated German version, whereby (after inverting) higher values indicate a higher quality of life.^30^

### Statistics

We first performed bivariate correlation analyses with the six PAHCO factors (T2) as independent and the number of steps (T3, T4) as dependent variables. The patients’ ages (in years), genders (male vs. female), body weights (body mass index), and disease severity (via FEV_1_) were treated as covariates.

Subsequently, we set up two structural equation models (SEM) in which the six-factor PAHCO assessment at T2 was multivariately regressed on each patient’s number of steps. The number of steps was, in turn, modelled on quality of life, complemented by direct, independent paths displayed by the control competence factors affecting the regulation and control of physical load.

We ran all computations with the software R (version 3.5.3), including the package lavaan. Robust maximum likelihood estimators (MLRs) and Satorra-Bentler scaled Chi-square statistics formed the basis for evaluating global model fits.^31^ As recommended,^32^ we reported standardized root mean square residual (SRMR), root mean square error of approximation (RMSEA), and comparative fit index (CFI). To interpret the magnitude of the values, we relied on guidelines displaying a good (SRMR ≤ 0.05, RMSEA ≤ 0.05, CFI ≥ 0.95) and acceptable/satisfactory (SRMR ≤ 0.10, RMSEA ≤ 0.08, CFI ≥ 0.90) model fit.^33,34^ The height of the coefficients *r* and *β* was determined as follows: small effect ≥ 0.10, moderate effect ≥ 0.30, and strong effect ≥ 0.50.^35^ We employed full information maximum likelihood (FIML) procedures to handle missing data.

## Results

### The role of PAHCO in PA behaviour and quality of life 6 weeks after rehabilitation (T3)

Five of the six PAHCO factors were univariately related to the number of steps at T3 (Table 2). Among the statistically significant variables, the association was strongest for the 6-minute walking test and lowest for PA-specific self-efficacy. Only the control of physical load factor did not display a statistically significant relationship. The associations of the six PAHCO factors with the quality of life outcome were all statistically significant, with the 6-minute walking test again showing the strongest coefficient and the control of physical load factor the lowest.

**Table 2.** Bivariate relationships of the PAHCO factors (T2) with the number of steps and quality of life 6 weeks (T3) and 6 months (T4) after rehabilitation.

PAHCO factor	Attributable sub-competence of PAHCO	Bivariate correlations with the outcomes at T3		Bivariate associations with the outcomes at T4	
		Number of steps	Quality of life	Number of steps	Quality of life
6-minute walking test	Movement competence	*r* = .58, *p* < .001**	*r* = .46, *p* < .001**	*r* = .54, *p* < .001**	*r* = .44, *p* < .001**
Control of physical load	Control competence	*r* = .07, *p* = .35	*r* = .25, *p* = .001**	*r* = .03, *p* = .61	*r* = .21, *p* = .006**
Affect regulation	Control competence	*r* = .20, *p* = .001**	*r* = .34, *p* < .001**	*r* = .21, *p* = .001**	*r* = .33, *p* < .001**
Self-efficacy	Self-regulation competence	*r* = .14, *p* = .04*	*r* = .38, *p* < .001**	*r* = .17, *p* = .008**	*r* = .32, *p* < .001**
Self-control	Self-regulation competence	*r* = .20, *p* = .001**	*r* = .36, *p* < .001**	*r* = .15, *p* = .02*	*r* = .34, *p* < .001**
Emotional attitude toward PA	Self-regulation competence	*r* = .23, *p* < .001**	*r* = .39, *p* < .001**	*r* = .21, *p* = .001**	*r* = .37, *p* < .001**
BMI (kg/m^2^)	Covariate	*r* = −.04, *p* = .51	*r* = .08, *p* = .18	*r* = −.08, *p* = .21	*r* = .03, *p* = .69
Gender (1 = male, 2 = female)	Covariate	*r* = -.02, *p* = .73	*r* = .03, *p* = .67	*r* = .01, *p* = .92	*r* = −.04, *p* = .49
Age (in years)	Covariate	*r* = −.10, *p* = .16	*r* = .06, *p* = .35	*r* = −.12, *p* = .09	*r* = .02, *p* = .79
Disease severity (via FEV_1_)	Covariate	*r* = .42, *p* < .001	*r* = .36, *p* < .001	*r* = .34, *p* < .001	*r* = .30, *p* < .001

*Note:* Details to the development of the PAHCO Questionnaire can be retrieved from Carl et al.,^19^ *p < .05. **p < .01.

SEM: structural equation model; PAHCO = physical activity-related health competence.

**Figure 3.**
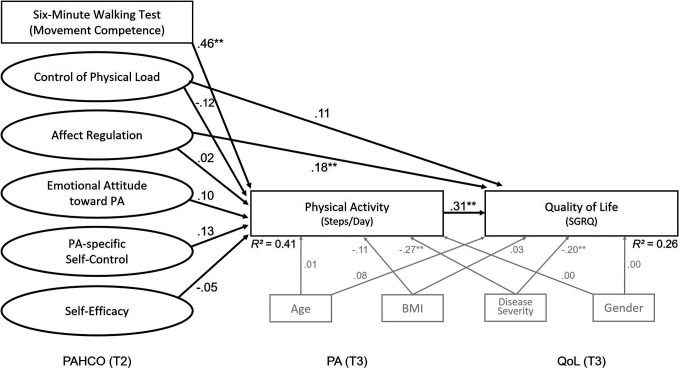
Visualization of the structural equation model for the prediction of the number of steps and patient’s quality of life 6 weeks (T3) after pulmonary rehabilitation. QoL: quality of life; SGRQ: Saint George’s respiratory questionnaire; PA: physical activity; PAHCO: physical activity-related health competence.

The SEM demonstrated a satisfactory model fit (CFI = 0.94, RMSEA = 0.05, SRMR = 0.07), which enabled us to interpret the paths of the model (see Figure 3). The 6-minute walking test dominated the predictive quality of the T2 PAHCO assessment for individuals’ number of steps at T3 (*β* = .46, *p* < .001). The remaining predictors showed no statistically independent association with the PA level (*p* > .19). The PA outcome, in turn, significantly predicted quality of life with a moderate effect size (*β* = .31, *p* < .001). The two sub-dimensions of control competence (*control of physical load* and *affect regulation*) contributed differently to predicting quality of life at T3. While the expected relationships were registered for affect regulation (*β* = .18, *p* = .02), the control of physical load factor displayed no statistically independent direct association with quality of life (*β* = .11, *p* = .17). The effects as described were found under consideration of the significant disease severity covariate (*β*_Steps_ = −.27, *p* < .001; *β*_SGRQ_ = −.20, *p* = .002).

### The role of PAHCO in PA behaviour and quality of life 6 months after rehabilitation (T4)

**Figure 4.**
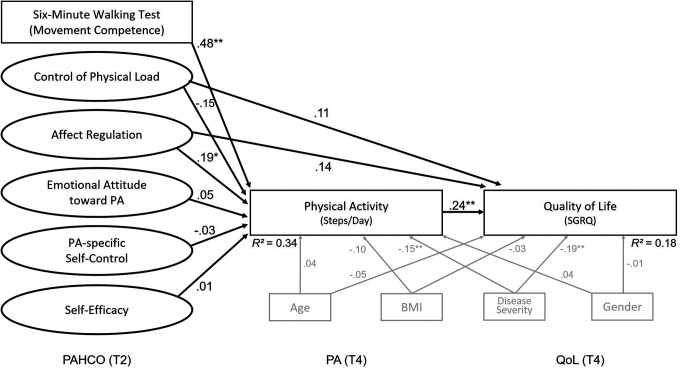
Visualization of the structural equation model for the prediction of the number of steps and patient’s quality of life 6 months (T4) after pulmonary rehabilitation. QoL: quality of life; SGRQ: Saint George’s respiratory questionnaire; PA: physical activity; PAHCO: physical activity-related health competence.

The bivariate pattern at T4 was comparable to the results at T3 (Table 2). The global fit of the SEM with the two outcomes at T4 was satisfactory (CFI = 0.95, RMSEA = 0.05, SRMR = 0.07). The multivariate model (for the SEM, see Figure 4) revealed significant associations for the 6-minute walking test (*β* = .48, *p* < .001) and the affect regulation factor (*β* = .19, *p* = .04). The other four PAHCO factors showed no statistically significant independent relationship with the number of steps at T4. Quality of life was, in turn, significantly predicted by the number of steps with a small-to-moderate effect size (*β* = .24, *p* = .002). Independent, statistically significant direct associations of the control competence factors with quality of life at T4 could not be found for either the affect regulation factor (*β* = .15, *p* = .11) or the control of physical load factor (*β* = .12, *p* = .23). The disease severity covariate also significantly influenced the predication of the two outcomes at T4 (*β*_Steps_ = −.15, *p* = .02; *β*_SGRQ_ = −.19, *p* = .004).

### Explanatory power of PAHCO: T3 vs. T4

The PAHCO assessment at T2 explained a total of 41.1% in the level of PA and 26.2% in individuals’ quality of life in the T3 model. Descriptively, the predictive quality of the PAHCO assessment was stronger (*ΔR*_Steps_^2^ = 0.07, *ΔR*_SGRQ_^2^ = 0.08) at T3 than T4. Nevertheless, the *R* ^2^ of the outcomes included in the T4 SEM was still fair, achieving values of 0.34 and 0.18, respectively.

## Discussion

This study demonstrated that the integrative PAHCO model holds a high predictive value for objectively measured PA in patients with COPD following PR. In participants of the STAR study,^25^ the PA-related competencies at the end of the rehabilitation stay explained about one third of the variance in daily steps 6 weeks and 6 months after PR. Contrary to our assumptions (Hypothesis a), not all sub-competencies of the PAHCO model contributed independently to the predictive value of the multivariate models. The SEM-based prediction of the PA volume was dominated by the 6-minute walking test as a proxy indicator of movement competence, while the influence remained after adjusting for covariates such as disease severity. The results from our multivariate model support previous studies that ascribe a central role to physical capacity in predicting the PA behaviour of patients with COPD.^36,13^ Given this finding, it would be valuable if exercise therapy primarily addressed the requirements underlying the 6-minute walking test. However, these requirements comprise not only endurance and strength capacities but also psychological (e.g., motivation, attitudes) and cognitive (e.g., pacing strategies) elements that co-determine the distance achieved.^37,38^

In line with this interpretation, the bivariate correlations suggested important further person-related determinants of PA. These analyses pointed to the relevance of all three sub-competences of the PAHCO model (movement, control, and self-regulation competence). More specifically, five out of six PAHCO items showed significant associations with the PA levels following PR. Only the control of physical load factor did not display any associations with the patients’ PA levels. The PAHCO items could help to understand under which conditions improvements in physical performance lead to increased PA. Long-term adherence to a physically active lifestyle is central for sustained health effects.^11^ Therefore, it is significant that T4 PA levels were partially predicted by the affect regulation factor as an indicator of control competence. This finding aligns with current research^39^ stating that the ability of individuals to gear PA toward positive affective reactions and subjective well-being enhances the probability of successful long-term adherence to regular PA. In summary, Hypothesis a, which had assumed independent predictive contributions from movement, control, and self-regulation competence factors, could only be confirmed partially.

Quality of life was significantly associated with individuals’ PA levels after rehabilitation. Our study participants showed improvements in the quality of life score from T0 to T2 (ΔSGRQ = −12), T3 (ΔSGRQ = −12) and T4 (ΔSGRQ = −8) that are highly clinical relevant.^40^ However, based on our analyses, we cannot quantify the exact contribution of the PA factor to these changes. Nevertheless, this finding supports similar research results from other patient groups, which showed that a change to a more physically active lifestyle is associated with a better quality of life, for example, in patients with multiple sclerosis,^41^ or recovering from cancer.^42^ On a broader level, this result highlights the importance of PA behaviour change as a key concept in PR.^43^

Quality of life was also influenced by a control competence factor at T3. Specifically, affect regulation showed an independent effect on quality of life beyond the mere PA volume at T3. However, such a path could not be registered for T4. The comparison of the predictive quality at T3 versus T4 demonstrated a slight decrease in the explanatory power of PAHCO for the PA level at T4 (Hypothesis c). Given the time lag between the PAHCO assessment and the final measurement of the PA behaviour (6 months after PR), it appears justified to highlight the sustainable role of PAHCO for a healthy, physically active lifestyle.

The pedometer-based behavioral intervention applied in the intervention group of the randomised, controlled STAR study aimed to increase PA after PR. However, the analyses (not published yet) showed no differences between control and intervention group in PA indicators and quality of life at T3 and T4. For this reason, we combined the two treatment groups in the statistical analysis without considering group assignment.

### Implications for physical activity promotion within pulmonary rehabilitation

In the context of PR, exercise is often still primarily understood as a biomedical intervention addressing physical capacity and physical fitness, whereas behaviour change is addressed by other interventional components such as education and self-management interventions.^10,44^ Biomedical exercise interventions improve exercise capacity and thereby help to improve aspects of movement competence as the most important PAHCO sub-competence. However, exercise therapy should also be tailored to other PAHCO aspects that affect PA behaviour. Affect regulation, for example, could be improved through the behaviour change technique *mood management*, which targets patients’ self-evaluations of their emotional status before and after exercise training or PA.^45^ The PAHCO action model highlights that exercise interventions in the context of PA promotion should enable ‘exercise’, ‘learning’, and ‘experience’ simultaneously, ideally in an interlocked way.^18^ Therefore, the PAHCO model and the related action model help therapists to set exercise therapy within a wider theoretical framework addressing both exercise capacity and the improvement of other PAHCO factors that influence PA.

### Limitations

This study has some limitations. First, the PAHCO questionnaire has been developed as a generic instrument. Even though the assessment has been specifically validated with COPD rehabilitants,^19^ some lung-specific facets of PAHCO with subjective and clinical value (e.g., disease-specific fears) may have not been captured by this instrument. Furthermore, the assessment has been complemented by a 6-minute walking test representing movement competence. It cannot be fully excluded that the strong relationship of this objective indicator with the accelerometer-based PA behaviour may partially result from different methodological approaches. This, in turn, means that movement competence could lose some of the explanatory power if also a questionnaire-based instrument had been chosen. Against this backdrop, caution must be warranted regarding the comparative weighting of the different PAHCO indicators as predictors of physical activity and quality of life. Future studies are strongly advised to use an instrument with the same format as the other PAHCO factors and, importantly, that does not have the character of a proxy indicator of a theoretical model component. More specifically, proxy indicator means that the 6-minute walking test is also influenced considerably by motivational and cognitive factors (e.g., pacing strategies) – determinants that can be partially attributed to self-regulation competence and control competence. Second, all participants were recruited in the same rehabilitation clinic, which limits the geographic generalizability of the findings. This circumstance is reinforced by the fact that PR on the international level often uses an outpatient format, whereas the present findings were gathered through an inpatient rehabilitation program. Simultaneously, the clinic specializes in pension-insured patients, which reduces the transferability of the present findings to patients with COPD in older age.

## Conclusion

The integrative PA-related Health Competence (PAHCO) model can predict PA levels in patients with COPD 6 weeks and 6 months after PR. The PA predictions were dominated by the 6-minute walking test – a proxy indicator of movement competence. In addition, affect regulation (representing control competence) had an independent influence on quality of life at the time point 6 weeks and on PA 6 months after PR. This study generates a better understanding of competence factors enabling patients with COPD to better initiate and maintain PA after PR. Therefore, the results help to optimize PA promotion in the context of PR.

## Supplemental Material

Supplemental Material, sj-docx-1-crd-10.1177_1479973121994781 - Towards a better understanding of physical activity in people with COPD: predicting physical activity after pulmonary rehabilitation using an integrative competence model Click here for additional data file. Supplemental Material, sj-docx-1-crd-10.1177_1479973121994781 for Towards a better understanding of physical activity in people with COPD: predicting physical activity after pulmonary rehabilitation using an integrative competence model by Johannes Alexander Carl, Wolfgang Geidl, Michael Schuler, Eriselda Mino, Nicola Lehbert, Michael Wittmann, Konrad Schultz and Klaus Pfeifer in Chronic Respiratory Disease

Supplemental Material, sj-docx-2-crd-10.1177_1479973121994781 - Towards a better understanding of physical activity in people with COPD: predicting physical activity after pulmonary rehabilitation using an integrative competence model Click here for additional data file. Supplemental Material, sj-docx-2-crd-10.1177_1479973121994781 for Towards a better understanding of physical activity in people with COPD: predicting physical activity after pulmonary rehabilitation using an integrative competence model by Johannes Alexander Carl, Wolfgang Geidl, Michael Schuler, Eriselda Mino, Nicola Lehbert, Michael Wittmann, Konrad Schultz and Klaus Pfeifer in Chronic Respiratory Disease

Supplemental Material, sj-docx-3-crd-10.1177_1479973121994781 - Towards a better understanding of physical activity in people with COPD: predicting physical activity after pulmonary rehabilitation using an integrative competence model Click here for additional data file. Supplemental Material, sj-docx-3-crd-10.1177_1479973121994781 for Towards a better understanding of physical activity in people with COPD: predicting physical activity after pulmonary rehabilitation using an integrative competence model by Johannes Alexander Carl, Wolfgang Geidl, Michael Schuler, Eriselda Mino, Nicola Lehbert, Michael Wittmann, Konrad Schultz and Klaus Pfeifer in Chronic Respiratory Disease
