# Supplementary material for: Towards a better understanding of physical activity in people with COPD: predicting physical activity after pulmonary rehabilitation using an integrative competence model
Source: Chron Respir Dis. 2021 Mar 11;18:1479973121994781. doi: 10.1177/1479973121994781 (PMC8718156; doi:10.1177/1479973121994781)
Supplement: Supplemental Material, sj-docx-1-crd-10.1177_1479973121994781 - Towards a better understanding of physical activity in people with COPD: predicting physical activity after pulmonary rehabilitation using an integrative competence model [file sj-docx-1-crd-10.1177_1479973121994781.docx]

**Supplementary File 1.** *Characteristics of the Sample at the End of Pulmonary Rehabilitation (T2) and Their Change Scores to T0/T1.*

| **Variable** | **Description at T2** | **Change Compared  to Baseline** |
| --- | --- | --- |
| FEV_1_ [%] | 58.38 ± 19.35 | 4.87 |
| Saint George’s Respiratory Questionnaire (SGRQ) Score | 40.20 ± 18.49 | -12.44 |
| Six-Minute Walking Test [m] | 520.14 ± 103.82 | 72.99 |

Note: Please see Table 1 in the main document for the baseline (T0/T1) values.
