# Supplementary material for: Towards a better understanding of physical activity in people with COPD: predicting physical activity after pulmonary rehabilitation using an integrative competence model
Source: Chron Respir Dis. 2021 Mar 11;18:1479973121994781. doi: 10.1177/1479973121994781 (PMC8718156; doi:10.1177/1479973121994781)
Supplement: Supplemental Material, sj-docx-2-crd-10.1177_1479973121994781 - Towards a better understanding of physical activity in people with COPD: predicting physical activity after pulmonary rehabilitation using an integrative competence model [file sj-docx-2-crd-10.1177_1479973121994781.docx]

**Supplementary File 2.** *Overview of the items of the Physical Activity-related Health Competence (PAHCO) model.*

| **Factor** | **Item** | ***Item Formulation*** |
| --- | --- | --- |
| Control of  Physical Load | CCPL1 | I am able to adjust my training effort well to my physical condition. |
|  | CCPL2 | I know how to use physical training to improve my endurance in the best possible way. |
|  | CCPL3 | If my muscles are tensed up, I know exactly how to counter this through physical activity. |
|  | CCPL4 | I can use my body signals (pulse, breathing speed) very well to gauge and regulate the amount of physical load. |
|  | CCPL5 | If I want to enhance my health by strengthening my trunk muscles (back, stomach), I am confident that I know the right exercises to do. |
|  | CCPL6 | I know what to pay attention to in relation to my body in order to avoid excess load or insufficient load. |
| Affect Regulation | AR1 | I am able to regulate my mood through physical activity. |
|  | AR2 | If I am feeling down, I can distract myself well through physical activity. |
|  | AR3 | I am well able to improve my depressed mood by exercising. |
|  | AR4 | I am well able to work off pent-up stress and inner tension through exercise. |
| Self-Efficacy | SE1 | I feel capable to perform physical activities that are challenging for me. |
|  | SE2 | I feel capable to perform highly challenging physical activities. |
|  | SE3 | I feel capable to perform even the most difficult sport activities. |
| Self-Control | SC1 | If I have planned to exercise, I generally follow through on this plan. |
|  | SC2 | I stick with my plan to do exercise and am not easily distracted from that plan. |
|  | SC3 | When I decide to do more exercise, I am very disciplined in implementing this plan. |
| Emotional Attitudes | ATEM1 | When I think of being physically active, I feel not/very relaxed. |
|  | ATEM2 | When I think of being physically active, I feel not/very content. |
|  | ATEM3 | When I think of being physically active, I feel not/very happy. |

Note: The latest version of the questionnaire contains five additional PAHCO factors. The current versions of the questionnaire are updated regularly and can be retrieved from a website of the local university (<https://www.sport.fau.de/das-institut/forschung/bewegung-und-gesundheit/forschungsprojekte/bgk/bgk-erstellung-eines-assessmentinstruments/>).
