# Supplementary material for: Towards a better understanding of physical activity in people with COPD: predicting physical activity after pulmonary rehabilitation using an integrative competence model
Source: Chron Respir Dis. 2021 Mar 11;18:1479973121994781. doi: 10.1177/1479973121994781 (PMC8718156; doi:10.1177/1479973121994781)
Supplement: Supplemental Material, sj-docx-3-crd-10.1177_1479973121994781 - Towards a better understanding of physical activity in people with COPD: predicting physical activity after pulmonary rehabilitation using an integrative competence model [file sj-docx-3-crd-10.1177_1479973121994781.docx]

**Supplementary File 3**. *Correlations between the different T2 Physical Actvity-related Health Comepentece (PAHCO) factors.*

|  | **Six-Minute Walking Test** | **Control of  Physical Load** | **Affect  Regulation** | **Self-Efficacy** | **Self-Control** | **Emotional  Attitude** |
| --- | --- | --- | --- | --- | --- | --- |
| **Six-Minute Walking Test** | - | 0.114 | 0.181 | 0.180 | 0.148 | 0.140 |
| **Control of  Physical Load** |  | - | 0.597 | 0.344 | 0.585 | 0.398 |
| **Affect  Regulation** |  |  | - | 0.421 | 0.588 | 0.605 |
| **Self-Efficacy** |  |  |  | - | 0.345 | 0.349 |
| **Self-Control** |  |  |  |  | - | 0.505 |

Notes: T2 = At the discharge of the inpatient stay of pulmonary rehabilitation. The standardized covariance (correlation) coefficients between the PAHCO factors in the structural equation model are displayed on the right side of the diagonal.
